# Oropharynx cancer after sleep apnea surgery

**DOI:** 10.1002/ccr3.5686

**Published:** 2022-04-08

**Authors:** Courtney Brooke Shires, Merry Sebelik

**Affiliations:** ^1^ 482972 West Cancer Center Germantown Tennessee USA; ^2^ 1371 Emory University Atlanta Georgia USA

**Keywords:** base of tongue, HPV, oropharynx cancer, sleep apnea surgery, tonsil, UPPP

## Abstract

Surgery can treat sleep apnea. An elderly male underwent lingual/palatine tonsillectomy for OSA. He was then found to have T3N2 p16+ squamous cell carcinoma. He is receiving chemoradiation. Recognition of occult malignancy in tonsillectomy specimens may facilitate early diagnosis and treatment for patients following sleep apnea surgery.

## Graphical abstract

We report a 73‐year‐old male who underwent palatine and lingual tonsillectomy for obstructive sleep apnea. A few months later, he was found to have advanced oropharynx cancer. It is likely that his cancer could have been discovered at an earlier stage if his oropharyngeal tissue had been sent for pathology evaluation at the time of his sleep apnea surgery.

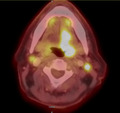

## 1 INTRODUCTION

Continuous positive airway pressure (CPAP) is the treatment of choice for moderate‐to‐severe obstructive sleep apnea (OSA). Approximately half of all the patients with OSA are unable to comply with prescribed CPAP therapy because of mask‐related problems, treatment‐related side effects, patient attitude, or perceived lack of benefit. Some patients who are intolerant to CPAP therapy may have anatomical differences that are amenable to surgery. For certain patients with OSA, surgery may be an alternative or adjunct to CPAP therapy.^1^

There are many different types of surgery for sleep apnea and snoring. Surgery may be a multi‐step process involving more than one procedure. Surgical options include uvulopalatopharyngoplasty (UPPP), radiofrequency volumetric tissue reduction (RFVTR), septoplasty and turbinate reduction, genioglossus advancement, hyoid suspension, maxillomandibular osteotomy (MMO) and advancement (MMA), palatal implants, weight loss surgery, laser‐assisted uvuloplasty (LAUP), and tracheostomy. Midline glossectomy and lingualplasty involve removing part of the back of the tongue. Making the tongue smaller can prevent airway blockage in some people with sleep apnea. These procedures are infrequent.^2^

Reduction glossectomy, which involves surgical debulking of the tongue, improves AHI (apnea hypopnea index) and ESS (endoscopic sleep study) scores significantly in 60% of patients.^3^The relatively new TORS (transoral robotic surgery) helps decrease the size of the base of the tongue (BOT) using operative debulking assisted by a surgical robotic. TORS BOT reduction has shown improvement in AHI and ESS scores in 68% of patients.^4^

While one indication for lingual tonsillectomy is lingual tonsil hypertrophy (LTH), which contributes to obstructive sleep apnea (OSA) in pediatric and adult patients,^4^ another indication is for squamous cell carcinoma of unknown primary (SCCUP) in the head and neck.^5^ A large number of SCCUP with no clinical or radiographical evidence of a primary tumor are most commonly found to have primaries in the palatine and lingual tonsils. More recently, performing palatine tonsillectomy and lingual tonsillectomy has resulted in finding the primary tumor in 75%–90% allowing patients to receive a decreased amount of more targeted radiation lessening side effects.^6^

The methods of lingual tonsillectomy include cold dissection, electrocautery, coblation, carbon dioxide (CO_2_) laser, and microdebrider.^7^, ^8^, ^9^ While these techniques destroy the lingual tonsils for benign indications, en bloc resection of the lingual tonsils is required in the workup of head and neck cancer, so that the pathologist can evaluate the tissue under a microscope.^10^ The presence of a surgical plane has been questioned in lingual tonsillectomy techniques. Joseph felt that there was a layer of fibrous tissue delineating the lingual tonsils from the tongue. Neither Joseph nor Dundar felt that there was a definite capsule of the lingual tonsil.^11^, ^12^ Lin found no clear demarcation between the lingual tonsils and the tongue musculature, although the change in tissue quantities was readily apparent to him.^13^ Son felt that deep to the lingual tonsils, a relatively avascular plane made up of connective tissue existed. This potential surgical plane is utilized in lingual tonsillectomy.^10^

Son described lingual tonsillectomy in 2016 using the robot. The lingual tonsil tissue was removed down to the muscle layer, starting medially, and dissecting laterally. A midline incision was made from the foramen cecum to anterior to the median glossoepiglottic fold. An incision was also made immediately posterior to the circumvallate papilla. The anterior medial edge of a side of the lingual tonsils was grasped and dissection was performed in the lateral posterior direction until the lingual tonsils were removed as one specimen for each side. Care was given to remove the lymphoid tissue keeping the underlying musculature intact.^10^

## 2 CASE REPORT

A 73‐year‐old male presented to a board‐certified Sleep Medicine Otolaryngologist for sinus and obstructive sleep apnea. The patient reported airway obstruction for 6 months, mostly consisting of nasal obstruction. His past medical history included atrial fibrillation and hypertension, and he had no smoking history. In early 2020, he underwent a septoplasty, lingual tonsillectomy, and palatine tonsillectomy. He was seen by the Otolaryngologist again in August 2020 with difficulty in breathing and wheezing. He was treated with antibiotics and steroids for chronic sinusitis and allergic rhinitis.

He underwent a CT scan of the maxillary sinus that did show some maxillary disease. He continued to have complaints of left ear pain and was sent for a CT scan soft tissue of neck. CT scan of the neck showed a 3.4 × 2.2 × 3.3 cm enhancing mass of the left base of the tongue/palatine tonsil. This did not cross midline or appear to extend into the hypopharynx. Multiple mildly prominent rounded left level 2 and 3 lymph nodes were present. There was a single left level 2 lymph node that measured 1.5 × 1 × 1.1 cm with illdefined margins concerning for early extracapsular spread.

On November18, 2020, he underwent direct laryngoscopy and biopsy. Biopsy of the left glossotonsillar sulcus showed P 16 positive squamous cell carcinoma.

**FIGURE 1:**
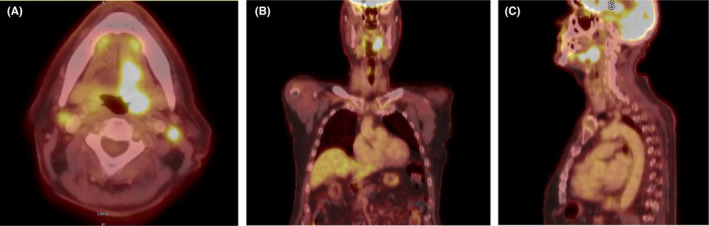
(A) Axial view of left oropharynx tumor and bilateral cervical adenopathy. (B) Coronal view of left oropharynx tumor. (C) Sagittal view of left oropharynx tumor

Positron emission tomography scan on December 8, 2020, showed a nodular enhancing mass involving the left tongue base and majority of the left aspect of the tongue, measuring up to 5 cm in maximum anteroposterior dimension (Figure 1A–C). The mass involved the left genioglossus muscle and likely the hypoglossus muscle. There was likely focal involvement of the left aspect of the mylohyoid as well. The mass did not cross midline. Posteriorly, the mass extended to the glossopharyngeal sulcus and left palatine tonsil. The posterior margin of the mass extended to within 2 mm of the left internal carotid artery. The margins of the mass were difficult to delineate. An intensely hypermetabolic left level 2A node measured 13 × 10 mm with likely invasion of the sternocleidomastoid indicating extranodal spread. There were also left level IIb and III nodes demonstrating FDG uptake. On the right, a well‐circumscribed level 2A node measured 11 × 9 mm and demonstrated focal FDG uptake. Other smaller right level IIB, III, and IV nodes also demonstrated FDG uptake.

For his T3N2 oropharynx cancer, he is receiving chemoradiation.

## 3 DISCUSSION

Our case was interesting, as lingual and palatine tonsillar tissue (in which squamous cell carcinoma arises) is significantly debulked after sleep surgery. There likely was residual tonsillar tissue after the initial sleep apnea surgery that may already have harbored an occult focus of malignancy.

Oropharyngeal squamous cell carcinoma arises in the lymphoid tissue of the base of tongue and palatine tonsils. Son described the correct plane in performing lingual tonsillectomy.^10^ In Son's resections of the lingual tonsils, there was a relatively less vascular or avascular area between the lingual tonsil and the underlying minor salivary glands and muscle tissue in all cases. Between the lingual tonsil and the submucosal muscular tissue at the base of the tongue, a distinct space or line was demonstrated in both benign and premalignant cases. The minor salivary gland and muscle were found to be intimately admixed especially in the superficial portion of the muscle. The presence of submucosal edema was felt to exaggerate the space, and presence of dysplasia associated with peritumoral lymphocytes infiltrate could obscure the space between the lingual tonsil and the underlying muscle. However, in all conditions, dissection between the two layers was feasible based on his histological study.^10^ This emphasized the importance of removing all of the lingual tonsil tissue in lingual tonsillectomy especially if the specimen was not going to be examined by Pathologist.

The presence of p16 positive staining in our patient's malignant tumor is an important surrogate for the etiology of SCC in his tonsillar tissue, as the phenotypic expression of HPV infection. Currently, more than 200 HPV genotypes have been identified.^14^ Mucosal HPV types are categorized into “high‐risk HPV” (HR‐HPV) and “low‐risk HPV” types according to their potential to cause malignancy in the cervix.^14^ Twelve HR‐HPV types have been labeled as oncogenic by the International Agency for Research on Cancer.^14^ Among the HR‐HPV types involved in head and neck cancer, HPV16 is, by far, the most common, with a prevalence >80% in patients diagnosed with oropharyngeal SCC. HPV18 causes a much smaller percentage (3%).^15^, ^16^, ^17^ The International Agency for Research on Cancer has estimated that approximately 31% of oropharyngeal SCC cases result from HPV.^18^

As mentioned, many previous studies have discussed the diminishing role of histopathological analysis of tonsillectomy specimens in benign tonsillectomy surgery, with evidence supporting only gross analysis.^19^ Currently, US hospitals do not have a standardized approach to processing tonsillectomy specimens. A survey of the American Academy of Otolaryngology Head and Neck Surgery in 2001 revealed a decreasing trend to send specimens for histologic examination.^20^ The most common reasons for this practice were cost savings, evidence based medical literature, patient age, absence of clinical suspicion for malignancy, risk factors (immunosuppression, various environmental exposures), and lack of suspicious gross findings of the tissue.^21^, ^22^, ^23^, ^24^, ^25^

In the adult patient population, the prevalence of unsuspected tonsillar malignancy in routine tonsillectomy specimens has been reported to be very low (0.03%; 1 case of squamous cell carcinoma out of 3904 cases).^19^ One study that analyzed 13,547 pediatric patients separately from adults showed a 0.044% prevalence of malignancy, with 2 (0.015%) being entirely unsuspected. A review of published studies involving 54,901 adult and pediatric patients showed a 0.087% incidence of tonsillar malignancy overall. Most patients with malignancy (88%) had recognized risk factors preoperatively, with only 0.015% of cases receiving an entirely unsuspected malignant diagnosis.^21^

However, given that most cases of malignant tonsil cancer are found after metastasis, there is concern about not performing routine histopathological analysis of tonsillectomy specimens.

The prevalence of HPV‐related oropharyngeal SCC has increased drastically in the past decade. Even though the current yield in screening seemingly benign tonsil specimens for carcinoma is remarkably low, over time, this may increase due to the rising incidence of HPV‐related oropharyngeal SCC.^26^ It may be necessary to weigh the previous low incidence of oropharynx SCC against the disadvantages of missing a cancer in the currently rising HPV population and the cost of caring for them.^27^

The literature and our experience suggest that most tonsillar malignancies present with suspicious clinical or gross findings; however, occult malignancies do occur. Although the incidence is very low, recognition of occult malignancy in tonsillectomy specimens may facilitate early diagnosis and treatment.^28^

## 4 CONCLUSIONS

Oropharynx cancer can occur even after sleep apnea surgery removing the lingual and palatine tonsils. In the rising era of HPV tumors, sending tonsillectomy specimens from sleep apnea surgery routinely for pathologic evaluation is warranted.

## CONFLICT OF INTEREST

The authors report no relevant financial disclosures related to this current work.

## AUTHOR CONTRIBUTIONS

Courtney B. Shires, MD, FACS, and Merry Sebelik, MD, collected the data, and wrote and edited article.

## ETHICAL APPROVAL

All issues related to ethics were taken into consideration throughout the study design and proposal and implemented during the research study itself. Informed consent was obtained, beneficence was made a top priority, and respect for confidentiality and privacy were upheld during the study and its various analysis and information assertation components.

## CONSENT

Written informed consent was obtained from the patient to publish this report in accordance with the journal's patient consent policy.

## Data Availability

Other desired data and material relevant to our study are available on request.
